# Profile of Immune Cells in Axillary Lymph Nodes Predicts Disease-Free Survival in Breast Cancer

**DOI:** 10.1371/journal.pmed.0020284

**Published:** 2005-09-06

**Authors:** Holbrook E Kohrt, Navid Nouri, Kent Nowels, Denise Johnson, Susan Holmes, Peter P Lee

**Affiliations:** **1**Department of Medicine, Division of Hematology, Stanford University, Stanford, California, United States of America,; **2**Department of Pathology, Stanford University, Stanford, California, United States of America,; **3**Department of Surgery, Division of Surgical Oncology, Stanford University, Stanford, California, United States of America,; **4**Department of Statistics, Stanford University, Stanford, California, United States of America; Sloan-Kettering Cancer InstituteUnited States of America

## Abstract

**Background:**

While lymph node metastasis is among the strongest predictors of disease-free and overall survival for patients with breast cancer, the immunological nature of tumor-draining lymph nodes is often ignored, and may provide additional prognostic information on clinical outcome.

**Methods and Findings:**

We performed immunohistochemical analysis of 47 sentinel and 104 axillary (nonsentinel) nodes from 77 breast cancer patients with 5 y of follow-up to determine if alterations in CD4, CD8, and CD1a cell populations predict nodal metastasis or disease-free survival. Sentinel and axillary node CD4 and CD8 T cells were decreased in breast cancer patients compared to control nodes. CD1a dendritic cells were also diminished in sentinel and tumor-involved axillary nodes, but increased in tumor-free axillary nodes. Axillary node, but not sentinel node, CD4 T cell and dendritic cell populations were highly correlated with disease-free survival, independent of axillary metastasis. Immune profiling of ALN from a test set of 48 patients, applying CD4 T cell and CD1a dendritic cell population thresholds of CD4 ≥ 7.0% and CD1a ≥ 0.6%, determined from analysis of a learning set of 29 patients, provided significant risk stratification into favorable and unfavorable prognostic groups superior to clinicopathologic characteristics including tumor size, extent or size of nodal metastasis (CD4, *p* < 0.001 and CD1a, *p* < 0.001). Moreover, axillary node CD4 T cell and CD1a dendritic cell populations allowed more significant stratification of disease-free survival of patients with T1 (primary tumor size 2 cm or less) and T2 (5 cm or larger) tumors than all other patient characteristics. Finally, sentinel node immune profiles correlated primarily with the presence of infiltrating tumor cells, while axillary node immune profiles appeared largely independent of nodal metastases, raising the possibility that, within axillary lymph nodes, immune profile changes and nodal metastases represent independent processes.

**Conclusion:**

These findings demonstrate that the immune profile of tumor-draining lymph nodes is of novel biologic and clinical importance for patients with early stage breast cancer.

## Introduction

Lymph node metastasis is well established among the strongest prognostic indicators of clinical outcome for patients with breast cancer [1–3]. The technique of sentinel lymph node (SLN) biopsy has been rapidly adopted over the past decade, as it accurately predicts axillary (nonsentinel) lymph node (ALN) metastasis and therefore identifies women who may be spared the morbidities of axillary dissection [4,5]. With the growing practice of SLN biopsy, new methods of lymph node analysis are being developed [3,6–8]. SLN evaluation by multiple hematoxylin and eosin stained sections (HES), immunohistochemistry (IHC), and most recently, RT-PCR for breast cancer-associated gene expression has increased metastasis detection by up to 42% [7,9]. Despite these technical advances, the prognostic significance of isolated tumor cells and RT-PCR–positive nodes remains inconclusive and highly debated.

Concurrent advances in pathological analysis of primary breast tumors have found infiltrating immune cells of prognostic significance [10,11]. Detailed histological analyses identified tumor-infiltrating T lymphocytes and dendritic cells, with diminished dendritic cell infiltration directly correlated with increased nodal metastasis and poor disease-free and overall survival [10,12–15]. Decreased circulating T lymphocyte populations have also been shown to correlate with poor overall survival [16]. Substantial evidence now exists showing impairment of the systemic and local immune response during breast cancer progression [10–17]. However, it is often overlooked that local tumor-draining nodes are the immunologically active sites where such immune responses, including tumor antigen presentation and lymphocyte activation, should develop. Impairment of the immune response is likely a critical step in lymph node invasion by tumor, and may precede microscopic metastasis detection. Indeed, a limited number of studies suggest that alterations in immune profile, including CD4 helper and CD8 cytotoxic T lymphocytes and CD1a dendritic cell populations, occur within the local nodes of breast cancer patients, although their clinical significance remains unknown [18–20]. Thus, we reasoned that immune profile analysis of tumor-draining nodes may be a more sensitive and earlier method of detecting metastasis, and may provide additional information on clinical outcome.

## Materials and Methods

### Study patients

Breast cancer patients aged 29–80 years treated at Stanford University Medical Center between February 1997 and January 1999 and found to have tumor-involved SLNs by multilevel HES or IHC were evaluated. Patients who subsequently underwent ALN dissection (ALND), as is standard clinical practice, with clinical outcome data available were selected. SLNs and ALNs were selected based on their designation as sentinel or axillary by the operative report; the majority of SLNs are ALNs based on their location within the breast at time of surgery. ALNs referred to in this study are all ALNs not designated as SLNs. For this reason, reference in this paper to ALNs and nonsentinel lymph nodes are synonymous. In surgical cases involving multiple SLNs and ALNs, one SLN (SLN series 1) and one ALN (ALN series 1) were arbitrarily selected by the Department of Pathology staff and represent the training set (*n* = 29). The Pathology staff member was blinded to the study design. As no randomization technique was employed, the training set selection process was by definition arbitrary rather than random. To test reliability and variance of immune profile, eight ALNs were selected from a single patient. For purposes of validating the training set, first, a second SLN and ALN were randomly selected (using the random selection function “sample” in R) for each individual within the training set; these represent training set SLN series 2 (*n* = 18; 11 of 29 patients had only a single SLN removed, which was included in SLN series 1), and training set ALN series 2 (*n* = 27; an additional ALN could not be retrieved for two of the original 29 patients). Second, a single ALN was randomly selected for all individuals within the test set (*n* = 48). SLNs and ALNs from patients within training set SLN series 2, training set ALN series 2, and the test set were randomly selected using the sample function in R [21]. As performed in prior studies to provide an average immune profile, ten control nodes—a single mesenteric node per control individual—were similarly examined from patients with benign disease without a history of malignancy or immune disorder [22–24]. All samples were collected from Stanford Department of Pathology Specimen Bank as coded specimens under a protocol approved by the Stanford University Medical Center Institutional Review Board.

All participants were untreated and without a history of cancer or immune disorder prior to breast cancer diagnosis and SLN biopsy. Following surgical management, patients received adjuvant therapy as determined by their medical and radiation oncologists. The duration of disease-free survival (DFS) was the time between initial diagnosis and first recurrence. All patients received SLN and ALN removal in conjunction with removal of primary tumor within 44 d of initial diagnosis. Initial diagnosis was performed by needle aspiration or core biopsy in the majority of cases. Final diagnosis was confirmed from the pathologic evaluation of the primary tumor from the lumpectomy specimen. The average difference between time of diagnosis and surgery was 12.3 d. We chose to use time of diagnosis rather than time of surgery to determine clinical outcome, as we were measuring the relationship between tumor and immune composition of local nodes versus the influence of surgery on outcome. All recurrences were based on documentation of local or systemic disease during a follow-up period of 5 y, after which data were censored. We recorded and verified patient, tumor, and lymph node characteristics [25].

### Immunostaining

Tissue sections, 3 μm thick, were cut from formalin-fixed, paraffin-embedded nodes. HES and IHC were performed after antigen retrieval using Biogenex Genomx i1000 (San Ramon, California, United States). Antibodies included anti-CD4 (1/20, Novacastro; Vector Laboratories, Burlingame, California, United States), anti-CD8 (1/25; Dako, Glostrup, Denmark), anti-CD1a (1/100, Dako), anti-AE1/AE3 (1/25, Biogenex), and, as secondary antibody, EnVision dextran kit (1/5, Dako). Optimal concentrations were determined, and tested in sample node sections. Double staining using 3′3′ diaminobenzidene, VIP (Vector Laboratories), and a light counterstain with Mayer's hematoxylin (Innogenex) was performed for lymphocyte populations of interest, with colocalization of tumor cells. Isotype-matched antibodies were used as negative controls. All slides for the respective antibody were stained in the same run.

Presence of metastasis was verified by HES and IHC on four sections per node by two blinded investigators trained in breast cancer pathology. Area of node occupied by each immune cell type and by tumor was determined through computerized image acquisition and analysis software (BLISS; Bacus Laboratories, Lombard, Illinois, United States). Prior image analyses determine cell count and area from an average of five to 20 high-power fields [10,14,15,20,26]. Using BLISS we acquired 160–4,130 sequential images at 200× of the entire lymph node section, which were sequenced together by Metamorph Imaging System (Universal Imaging, Sunnyvale, California, United States). Objectives were calibrated to transform image pixels to microns. Control nodes were examined to standardize thresholds of each stain for cell of interest. Using an automated Metamorph script, standardized thresholds were applied with Metamorph log set to record areas occupied by cell of interest, tumor, and of entire node for all samples, thus minimizing any potential operator bias.

### Statistical analysis

Univariate and multivariate analyses including logistic regression tested predictive capacity of patient characteristics. Immune profiles of patients with and without nodal metastasis, and with and without disease recurrence, were compared by Wilcoxon rank sum test. F-test for immune profile equality of variance analysis was used to determine variance between nodes from a single patient versus nodes from different patients with similar characteristics [21]. Variance was also calculated for pairs of ALNs with similar tumor status (either both tumor-free, or both tumor-involved) from the same patient, versus variance for pairs of ALNs with discordant tumor status (one tumor-free and one tumor-involved) from the same patient. ALN series 1 immune profile's sensitivity and specificity in predicting disease recurrence were determined from receiver-operating-characteristic (ROC) curves based on the ALN immune profile of patients with versus without disease recurrence from the training set. ALN series 1 immune profile thresholds were applied to SLN series 2, ALN series 2 and the test set with statistical comparison by *X* ^2^ test. We constructed Kaplan-Meier (KM) life-table curves for DFS, with permuted log-rank test comparisons, as the sample size was limited. The training set was stratified for KM curves by ALN series 1 and 2 immune profiles, established from ROC curves applied to ALN series 1, to test prediction of DFS. Nodal thresholds from the training set ALN series 1 were also applied to the test set in KM curves compared by permuted log-rank tests. For analyses involving ALNs from all participants, the only available ALN from the test set was selected. However, as the learning set had two possible ALNs (series 1 and series 2), the sample function in R was used to randomly select one of the two ALNs from the learning set by random number generation. Finally, immune profile and clinicopathologic characteristics significant by univariate analyses among all 77 patients, those with T1 tumors, and/or those with T2 tumors were entered into a Cox proportional hazards model. Two-sided *p* < 0.05 was considered a statistically significant difference. For analyses we used R statistical package [21,27,28].

## Results

### Patient, Primary Tumor, and Lymph Node Characteristics

Characteristics of the training set (29 patients) are shown in Table 1. Of 29 SLN metastases in SLN series 1, all were tumor-involved, five contained isolated tumor cells, 11 contained micrometastases, and 13 contained macrometastases. Of 18 SLNs in series 2, nine were tumor-involved, three contained micrometastases, and six contained macrometastases; 16 individuals had positive ALNDs. Of 29 arbitrarily selected series 1 ALNs, nine were found to be tumor-involved, with seven of the 20 tumor-free ALNs selected from patients with positive ALNDs (ALNs other than the one selected for series 1 were found to be tumor-involved) (Figure S1). Of 27 randomly selected series 2 ALNs, seven were tumor-involved (Figure S2). Recurrent disease developed in 11 of 29 patients with 5 y of follow-up; two of 11 recurrences (18%) occurred at a distant site, and ten of 11 developed locoregional relapse (91%), with one patient at time of relapse found to have both local and distant disease.

**Table 1:**
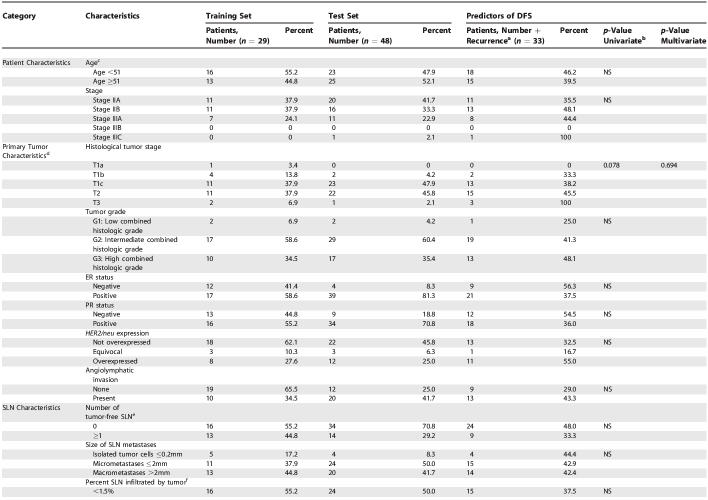
Patient, Primary Tumor, and Lymph Node Characteristics

**Table 1:**
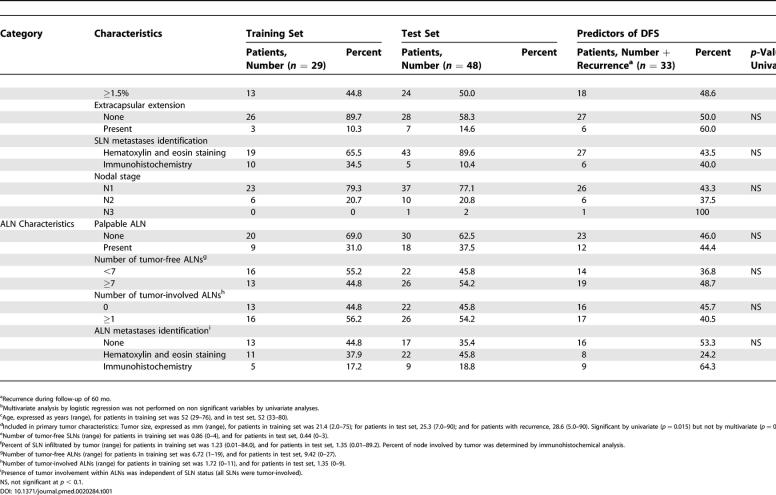
Continued

Test set (48 patients) clinicopathologic characteristics are shown in Table 1. All patients had a tumor-involved SLN biopsy, with four containing isolated tumor cells, 24 containing micrometastases, and 20 containing SLN macrometastases. Recurrent disease developed in 22 (45.8%) of 48 patients during follow-up of 5 y; 14 of 22 occurred at distant sites, seven developed locoregional relapse, and one recurred both at a distant site and locally. ALNs selected from eight (36.3%) of 22 patients with disease recurrence were tumor-involved (Figure S3). Of the 26 ALNs selected from patients without recurrent disease, nine (34.6%) were tumor-involved.

Among all patients from both training set and test set (*n* = 77), only tumor size significantly correlated with disease recurrence (*p* = 0.015). Among patients with only T1 tumors (*n* = 41), percent tumor involvement in the SLN correlated with disease recurrence more closely than all other clinicopathologic characteristics, (*p* = 0.057). Likewise, among patients with only T2 tumors (*n* = 33), size of SLN metastasis correlated with disease recurrence more closely than all other clinicopathologic characteristics (*p* = 0.041).

### Alterations in Immune Profile of Tumor-Draining Lymph Nodes

**Figure 1:**
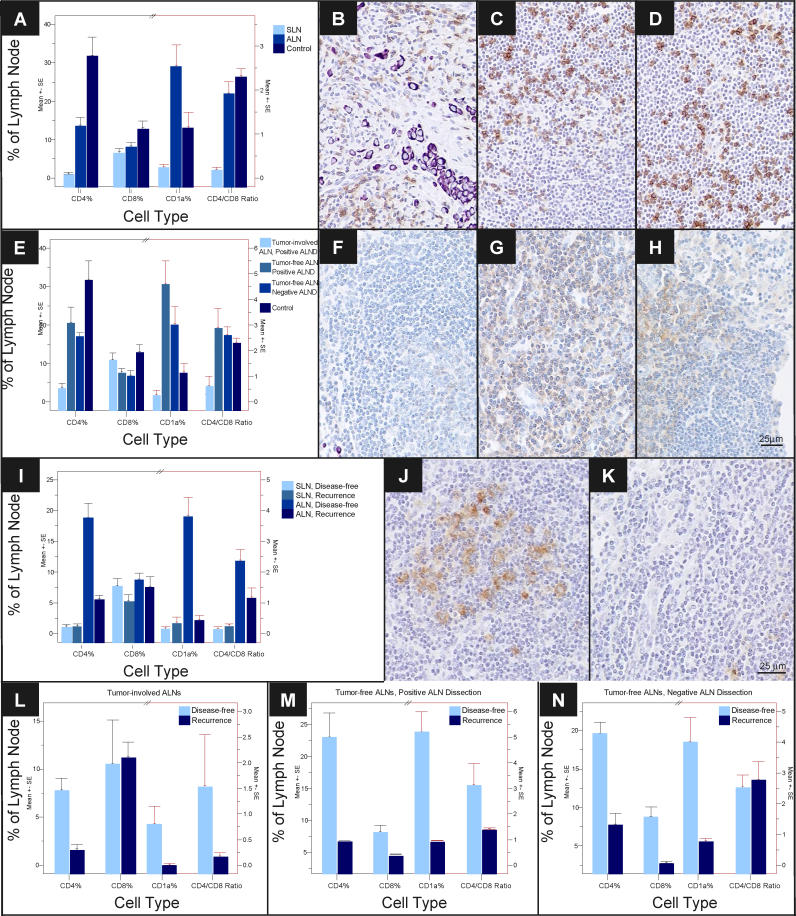
Lymph Node Profile of Sentinel and Axillary Lymph Nodes Mean and standard error of CD4 and CD8 T cell, CD1a dendritic cell populations as percent of lymph node, and CD4:CD8 cell ratio are shown for (A) SLN (*n* = 29), ALN (*n* = 29), and control lymph nodes (*n* = 10); (E) tumor-involved ALNs (*n* = 9), tumor-free ALNs (*n* = 7) from patients with a positive ALND, tumor-free ALNs from patients with a negative ALND (*n* = 13), and controls (*n* = 10); (I) SLNs and ALNs stratified by disease recurrence during 5 y of follow-up (11 of 29 with recurrent disease); (L) tumor-involved ALNs stratified by disease recurrence (*n* = 9); (M) tumor-free ALNs from patients with a positive ALND stratified by disease recurrence (*n* = 7); and (N) tumor-free ALNs from patients with a negative ALND stratified by disease recurrence (*n* = 13). Representative 200× images of lymphocyte population (brown staining) and infiltrating tumor (purple staining) by IHC, including CD8 T cells in (B) SLNs, (C) ALNs, and (D) controls; (F) CD4 T cells in tumor-involved ALNs, (G) tumor-free ALNs from patients with a positive ALND, (H) tumor-free ALNs from patients with a negative ALND; and (J) CD1a dendritic cells in ALNs from patients disease-free versus (K) patients who developed recurrence.

To determine whether tumor-draining lymph nodes from patients with breast cancer are different immunologically than lymph nodes from control individuals, we initially analyzed one SLN and one ALN from each of 29 breast cancer patients (training set, Table 1) by IHC for CD4 T cell, CD8 T cell, and CD1a dendritic cell populations (Figure 1). We found significant differences in CD4 and CD1a populations between SLN, ALN, and control nodes (Figure 1A). While control nodes contained the highest percentages of CD4 and CD8 T cells, ALNs contained the highest percentage of CD1a cells (Figure 1A). The magnitude of CD4 population decrease from control nodes to SLNs was over 10-fold greater than the CD8 decreases between these nodes. SLNs also displayed significant decreases in CD1a cells. Interestingly, CD1a cells were elevated in ALNs even above controls. To determine if tumor invasion is a prerequisite for alterations in immune profile, training set SLNs and ALNs were grouped together as tumor-free or tumor-involved, which revealed dramatic differences in CD4 and CD1a populations and CD4:CD8 ratio based on tumor status (Table 2). Furthermore, training set ALNs (Figure S1) were stratified as tumor-involved (*n* = 9), tumor-free from an individual with positive ALND (*n* = 7), or tumor-free from an individual with negative ALND (*n* = 13). CD4 and CD1a cells were significantly decreased in tumor-involved ALNs (Figure 1E). Intriguingly, CD4 populations were decreased even in tumor-free ALNs (Figure 1E), suggesting that these changes are not merely a reflection of tumor invasion. In contrast, tumor-free ALNs showed significant increases in CD1a cells, which is more dramatic in those from individuals with a positive ALND (Figure 1E). Analysis of percent of node involved by tumor and magnitude of CD4, CD8, or CD1a changes did not show a statistically significant relationship. These observations argue against a simple linear relationship between immune alterations and tumor invasion, but suggest that dynamic changes in the immune profile within tumor-draining lymph nodes may in fact precede tumor invasion.

**Table 2:**
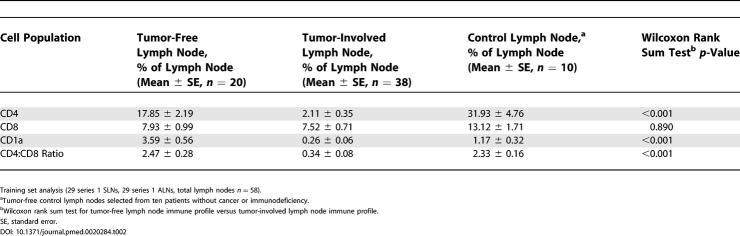
Immune Profile and Nodal Status

### Relationship between SLN Immune Profile and Axillary Metastasis or DFS

We investigated whether a relationship exists between SLN immune profile and ALN metastasis or DFS. While SLN CD4 populations and CD4:CD8 ratio demonstrated a trend toward an association with axillary metastasis (Table 3), CD8 and CD1a populations showed no such relationship. When SLN immune profile was analyzed for DFS, CD8 populations showed a trend; however, all other cell populations showed no statistically significant relationship with survival (Figure 1I; Table 3).

**Table 3:**
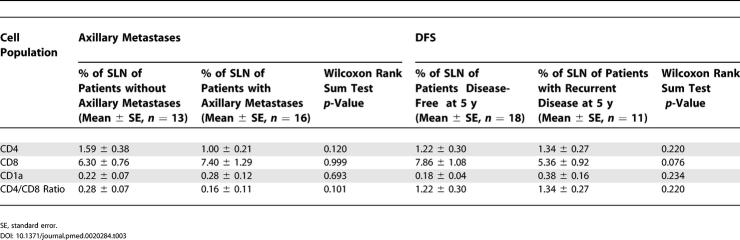
SLN Immune Profile and Clinical Outcome

### ALN Immune Profile and Disease-Free Survival

In contrast to SLNs, which exhibited similar immune profile changes in all 29 training set individuals, ALN CD4 and CD1a populations showed significant differences between patients with recurrence versus those disease-free at 5 y (*p* < 0.001) (Figure 1I and 1K; Table 4). Furthermore, associations between disease recurrence and changes in ALN CD4 and CD1a populations were independent of nodal metastasis or ALND status (Figure 1L–1N). Among patients with disease recurrence, degree of decrease in CD4 T cell and CD1a dendritic cell populations was similar (greater than 4-fold) among tumor-involved ALNs and tumor-free ALNs from either positive or negative ALNDs. These findings support a direct relationship between ALN immune profile and disease-free survival—even within these arbitrarily selected ALNs (series 1), regardless of nodal and locoregional metastasis status.

**Table 4:**
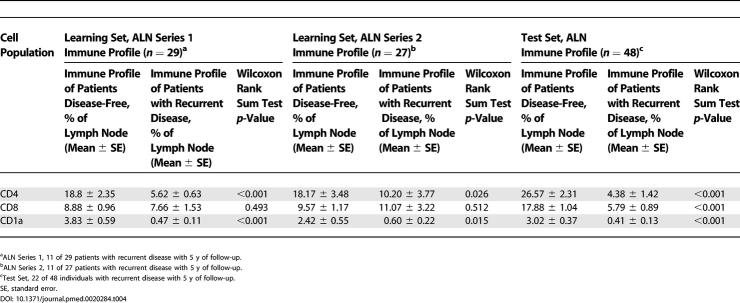
ALN Immune Profile and DFS

**Figure 2:**
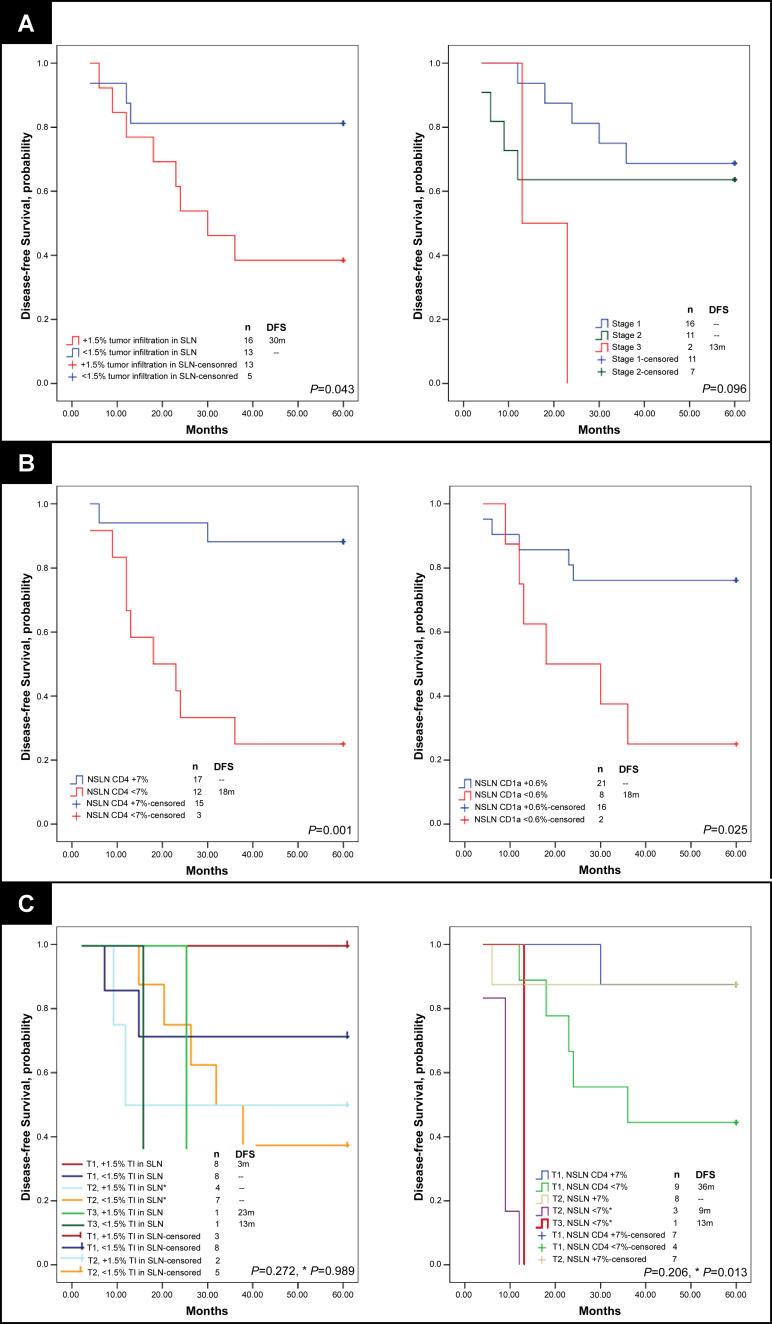
Disease-free Survival Analysis of Women with Breast Cancer According to Immune profile Characteristics, Learning Set ALN Series 2, and Test Set KM curves are shown for (A) median DFS applied to the learning set ALN series 2 (*n* = 27) and test set (*n* = 48) according to size of CD4 T cell and CD1a dendritic cell populations within learning set ALN series 2 (second, randomly selected ALN per individual); (B) DFS stratified by size of CD4 T cell and CD1a dendritic cell populations within test set ALNs; and (C) DFS applied to the learning set (*n* = 29) and test set (*n* = 48) according to size of ALN CD4 T cell and ALN CD1a dendritic cell populations. Thresholds for ALN CD4 T cell and ALN CD1a dendritic cell populations were determined by ROC curves as applied to the learning set (ALN series 1). Median duration of DFS are indicated; – indicates a median DFS greater than follow-up period, 5 y. Of 29 individuals in learning set ALN series 1, 11 had recurrent disease, and of 27 individuals in learning set ALN series 2, 11 had recurrent disease. Of 48 individuals in the test set of ALNs, 22 had recurrent disease. For ALN selection from the learning set (C), a single ALN was randomly selected from series 1 or series 2 per individual. Adjusted *p*-values were determined by the permuted log-rank statistic for comparison of DFS between groups.

To expand on the applicability of these findings, we randomly selected a second ALN from 27 of the 29 individuals in the training set (series 2, Figure S2). Immune profile thresholds determined from ROC curve analysis for maximal predictive accuracy among the training set ALN series 1 were applied to these additional 27 ALNs. Stratification of the training set into favorable and unfavorable prognostic groups for CD4 and CD1a populations was highly significant as displayed in KM curves of DFS (*p* = 0.005 and *p* = 0.007, respectively) (Figure 2A; Table 4).

**Figure 3:**
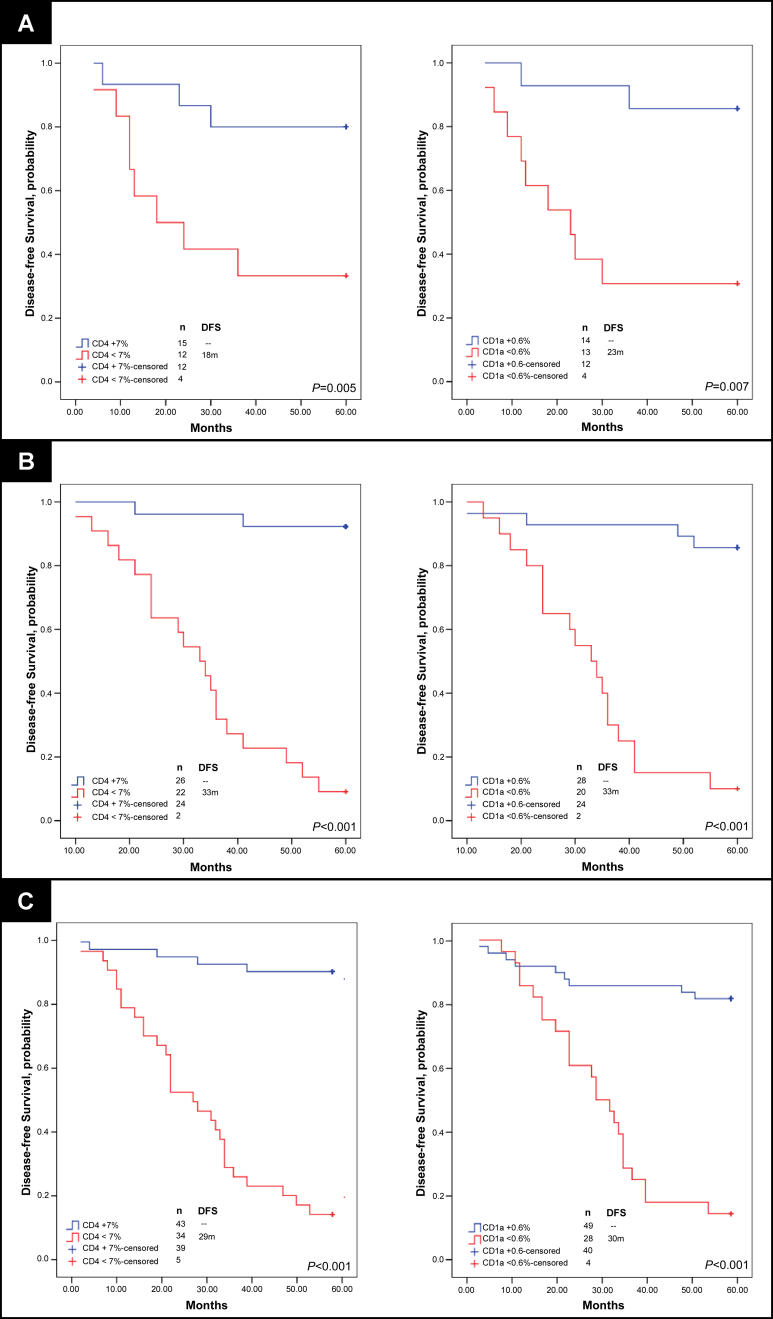
DFS Analysis of Women with Breast Cancer According to Tumor and Immune profile Characteristics, Learning Set ALN Series 1 KM curves are shown for (A) median DFS applied to the learning set, *n* = 29, according to percent of SLN occupied by infiltrating tumor (determined by IHC), and stratified by tumor stage; (B) DFS according to size of CD4 T cell and CD1a dendritic cell populations within learning set ALN series 1 (first, arbitrarily selected ALN per individual); and (C) DFS stratified both by percent of SLN infiltrated by tumor and tumor stage, and by both axillary node CD4 T cell population and by tumor stage. A comparison of survival by all subgroups and a separate comparison of stratified T2 alone are included (* in [C]). Thresholds for percent tumor infiltration within SLN, ALN CD4 T cell, and ALN CD1a dendritic cell populations were determined by ROC curves as applied to the learning set (SLN and ALN series 1). Median duration of DFS are indicated; – indicates a median DFS greater than follow-up period, 5 y. Of 29 individuals, 11 had recurrent disease. Adjusted *p-*values were determined by the permuted log-rank statistic for comparison of disease-free survival between groups. TI, tumor infiltration.

Additional comparison of immune profile and patient characteristics within the training set demonstrated ALN CD4 T cell and CD1a dendritic cell populations had superior predictive capacity of DFS (*p* = 0.001 for both) compared to the degree of tumor involvement in SLNs and ALNs or to primary tumor size, by ROC curve analyses (*p* = 0.039, *p* = 0.102, and *p* = 0.072) (Figure S4). KM curves indicated significant stratification of DFS by percent of tumor involvement in SLN series 1, tumor stage, and ALN CD1a and CD4 populations (Figure 3) (*p* = 0.043, *p* = 0.096, *p* = 0.001, and *p* = 0.025). Patient stratification by both ALN CD4 T cell population and tumor stage predicted DFS equally as well as, if not better than, the most statistically significant clinicopathologic characteristics (tumor stage and percent of tumor involvement in the SLN) (Figure 3C).

### Intra-Individual Versus Inter-Individual Variance in Lymph Node Immune Profile

To more fully address the issue of internodal variance in immune profile from a single individual, we analyzed the immune profiles of eight randomly selected ALNs from a single patient. The variance of these nodes was compared to the variance of nodes from different individuals with similar patient characteristics, including similar recurrent disease state (*n* = 66). Equality of variance testing illustrated intra-individual homogeneity between nodes relative to inter-individual nodal variance for CD1a, CD4, and CD8 (F_[65,7]_-statistics of 24.65, 26.89, and 10.23; corresponding significances *p* < 0.001, *p* < 0.001, and *p* = 0.002, respectively).

### Validation of the Predictive Capacity of ALN Immune Profile

**Figure 4:**
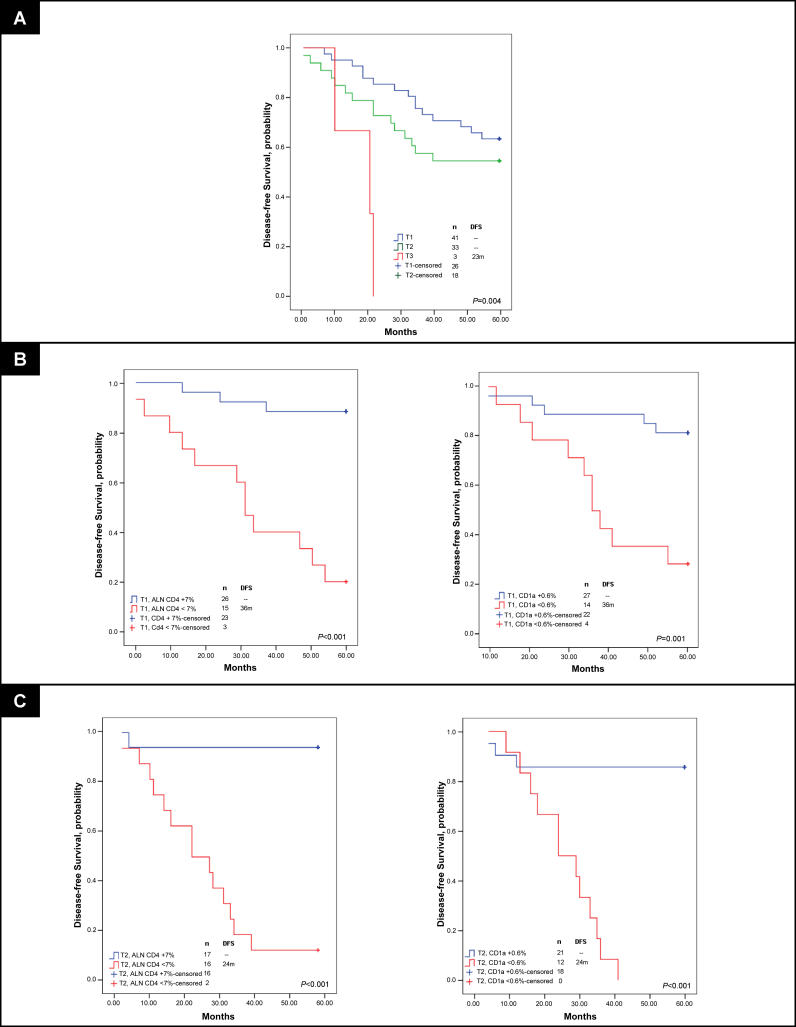
DFS Analysis of Women with Breast Cancer According to Tumor Stage, T1 and T2, and Immune Profile Characteristics, Learning and Test Sets KM curves are shown for (A) median DFS applied to the learning set (*n* = 29) and test set (*n* = 48) according to tumor stage; (B) DFS stratified by size of ALN CD4 T cell and ALN CD1a dendritic cell populations among individuals with T1 tumors; and (C) DFS stratified by size of ALN CD4 T cell and ALN CD1a dendritic cell populations among individuals with T2 tumors. Thresholds for ALN CD4 T cell and ALN CD1a dendritic cell populations were determined by receiver-operating-characteristic curves as applied to the learning set (ALN series 1). Median duration of DFS are indicated; – indicates a median DFS greater than follow-up period, 5 y. Of 77 individuals, 33 had disease recurrence. Of 41 from individuals with T1 tumors, 15 had recurrent disease. Of 33 individuals with T2 tumors, 15 had disease recurrence. For ALN selection from the learning set, a single ALN was randomly selected from series 1 or series 2 per individual. Adjusted *p*-values were determined by the permuted log-rank statistic for comparison of DFS between groups.

To further validate the predictive capacity of ALN immune profile for DFS in breast cancer, we analyzed one randomly selected ALN from an additional 48 patients (test set, Table 1), 22 of which developed recurrent disease in 5 y. Thresholds determined by ROC curves from the training set series 1 were applied to the test set data, which demonstrated highly significant stratification of favorable and unfavorable risk of recurrent disease (KM curves of DFS and permuted log-rank tests significant with *p* < 0.001 for both CD4 and CD1a populations; Figure 2B). Final comparison of the predictive strength of ALN immune profile relative to the most predictive clinicopathologic characteristics was performed for all patients with recurrence status available (single ALN selected randomly from learning set series 1 or series 2, *n* = 27; and ALN test set, *n* = 48; total ALNs *n* = 77; Figure 2C). Of 77 patients analyzed, 33 developed recurrent disease during the follow-up period. Among all patients from both training set and test set, only tumor size significantly correlated with disease recurrence (*p* = 0.015). KM curves of DFS stratified by ALN CD4 population and ALN CD1a population demonstrate superior risk stratification for recurrence by immune profiling compared to tumor size (*p* < 0.001, *p* < 0.001, and *p* = 0.004, respectively; Figures 2C and 4A).

### Strength of ALN Immune Profile as Predictors of DFS in Early Stage Patients (T1 and T2 Tumors)

The predictive value of ALN immune profile was particularly striking in early stage breast cancer patients (with T1 and T2 tumors) (Figure 4). Among the learning set, patients with T2 tumors and ALN CD4 population less than 7.0% had a median duration to recurrence of 9 mo and five-year DFS rate of 0%, versus a median DFS greater than follow-up period of 5 y and DFS rate of 88% for those with T2 tumors and ALN CD4 population of 7.0% or above (*p* = 0.01) (Figure 4C). By immune profiling of the entire study population (*n* = 77), median DFS for the unfavorable CD4 and CD1a profiles among 33 patients with T2 tumors were both 24 mo with DFS rates of 13% and 0.0%, respectively. In contrast, favorable ALN CD4 and CD1a profiles portended DFS rate of 94% and 86%, respectively. DFS according to CD4 and CD1a immune profiles was superior to all other clinicopathologic characteristics, the most predictive characteristic being size of SLN metastasis (permuted log-rank test, ALN CD4, *p* < 0.001; ALN CD1a, *p* < 0.001; and size of SLN metastasis, *p* = 0.03). Furthermore, ALN immune profiles of CD4 or CD1a cells were significantly superior to prognostic capacity by amount of local metastatic tumor burden (number of tumor-involved ALNs, *p* > 0.05) among patients with T2 tumors.

For patients with T1 tumors, we similarly determined the best current clinicopathologic predictor of disease recurrence in 41 patients with T1 tumors among our study population. This characteristic, percent of tumor involvement within the SLN, was an inferior predictor to immune profiling by ALN CD4 and CD1a (permuted log-rank test, percent tumor involvement in SLN, *p* = 0.049; CD4, *p* < 0.001; and CD1a, *p* = 0.001; Figure 4B). By ALN immune profiling among patients with T1 tumors, median DFS for the unfavorable CD4 and CD1a profiles were both 36 mo with DFS rates of 20% and 29%, respectively. Favorable ALN immune profiles portended a significantly more favorable DFS rate of 88% and 81% for CD4 and CD1a among patients with T1 tumors. Thus, for patients with T1 tumors, DFS according to CD4 and CD1a immune profiles was also superior to current clinicopathologic characteristics, including the number of tumor-involved ALNs (*p* > 0.05).

### Relationships between Immune Profile and Metastasis in SLN and ALN

To address potential mechanisms of immune changes in breast cancer-draining lymph nodes, we further explored the dependence of immune profile changes on nodal tumor metastasis in SLNs and ALNs. Immune profile thresholds determined from ROC curve analysis of training set series 1 lymph nodes (CD4 at 7%, CD1a at 0.6%) were applied to SLNs from training set series 2 and ALNs from training set series 2 and the test set. While all of the series 1 SLNs were tumor-involved, only 50% of the series 2 SLNs were involved, making such an analysis possible for both SLN and ALN. Lymph nodes were segregated based on immune profile changes and nodal metastasis (Table 5). Among the 18 SLNs, all nine (100%) tumor-involved SLNs showed decreased percentages of CD4 cells, and 77.8% showed decreased percentages of CD1a cells. Conversely, 81.8% and 77.8% of SLNs with relatively normal percentages of CD4 cells and CD1a cells, respectively, were tumor-free. *X* ^2^ testing for CD4 and CD1a, with *p*-values of less than 0.001 and 0.017, respectively, demonstrate the strength of relationship between tumor involvement and immune profile in SLNs.

**Table 5:**
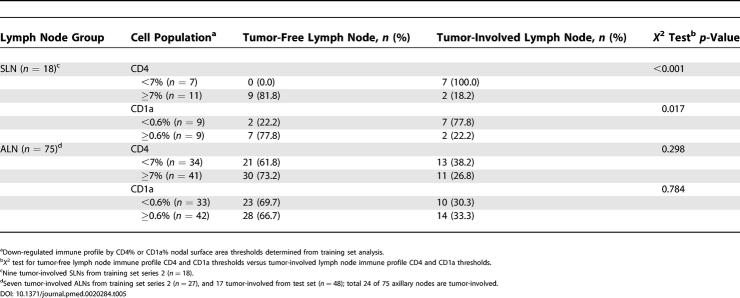
Sentinel and Axillary Lymph Node Immune Profile and Nodal Metastases

Importantly, ALN analysis of 75 nodes from training set series 2 and the test set, 24 of which were tumor-involved, did not demonstrate a similar effect of nodal tumor status on nodal immune profile (Table 5). Of the 24, 11 (46%) tumor-involved ALNs exhibited preserved CD4 percentages, and 14 (58%) exhibited preserved CD1a percentages. Furthermore, of 51 tumor-free ALNs, 21 (41%) and 23 (45%) exhibited decreased percentages of CD4 or CD1a cells, respectively. Hence, among these ALNs, no statistically significant association was found between decreased CD4 or CD1a populations and nodal tumor involvement (*p*-values 0.298 and 0.784, respectively). To address the dependence of ALN immune profile on nodal tumor status, we directly compared the immune profiles of series 1 and series 2 ALNs from the same patient. Of 27 paired ALNs, seven were discordant (one tumor-involved and one tumor-free), allowing us to address whether nodal metastasis is the dominant cause of ALN immune profile changes within individuals. Interestingly, the variance between discordant ALN pairs from the same patients was the same or even less than the variance between concordant ALN pairs (both tumor-involved or both tumor-free) (Table 6). This further supports the possibility that ALN immune profile change is driven by a separate process from nodal metastasis.

**Table 6:**
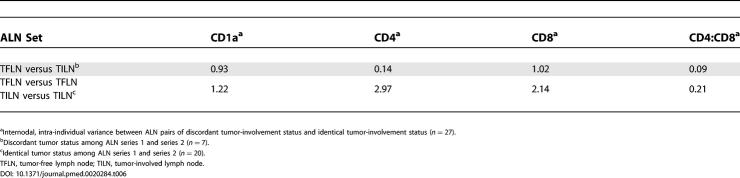
Intra-Individual ALN Immune Profile Variance

Finally, the independent predictors of DFS are shown in Table 7. The most significant independent predictors were percent of CD1a and CD4 cells in the ALN (hazards ratios of 0.42 and 0.93, respectively). Tumor size displayed a trend with recurrence (although not significant at *p* < 0.05), with a hazards ratio of 1.18. Neither the percent of tumor within the analyzed ALN, nor the size of tumor metastasis within the SLN, were associated with DFS by Cox proportional hazards modeling. These findings point to the intriguing possibility that immune profile changes and nodal metastasis may be independent processes in ALN. This is in contrast to SLN, in which immune profile changes appear dependent on nodal metastasis. Importantly, our data show that ALN immune profile—not SLN immune profile (see Table 3) or ALN metastasis (Table 8)—predicts DFS in breast cancer.

**Table 7:**
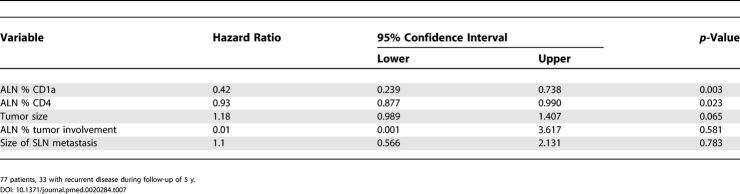
Cox Proportional Hazards Model for DFS

**Table 8:**
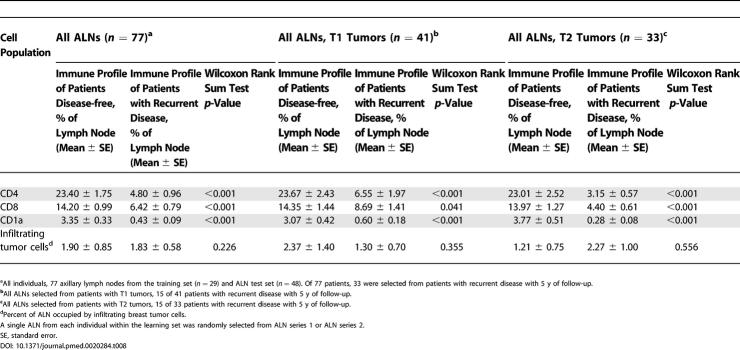
ALN Immune Profile, Tumor Stage, and DFS

## Discussion

It is now widely accepted that the status of tumor-draining lymph nodes significantly predicts clinical outcome in breast cancer. However, current clinical practice involves only histological examination of such nodes for the presence or absence of tumor, largely ignoring the immunological nature of lymph nodes in cancer. As the systemic immune response is clearly influenced by tumor progression, immune profile changes in early sites of immune system-cancer interactions, i.e., tumor-draining nodes, may represent a sensitive indicator of tumor metastasis [10,16,22]. More significantly, the nature of such immunological changes may provide additional biological and prognostic information. In this study, we analyzed the lymph node immune profiles in 77 breast cancer patients with tumor-involved SLNs, 42 of which had tumor-positive ALNDs. Importantly, in 5 y of follow-up, 33 patients had disease recurrence, allowing us to correlate nodal immune profile with clinical outcome. Four patients had SLNs containing isolated tumor cells (0.2 mm or smaller) detected by only IHC—these patients developed disease recurrence, supporting the clinical significance of IHC-only positive SLNs [6,7,29]. As in other studies, mesenteric nodes from patients with benign disease were used as comparisons, since axillary nodes are rarely excised for nonmalignant conditions [22–24]; immune profile of control nodes paralleled literature standards [23,24]. Importantly, new computer-based imaging techniques provided high-resolution image acquisition of the entire nodal surface. We acquired a total of 160–4,130 images (200× magnification) per nodal section, while prior studies based their results on only 5–20 images per section [10,14,15,20,26]. By such detailed, automated analysis of SLNs and ALNs, we identified unique patterns in the degree of CD4 helper T cell, CD8 cytotoxic T cell, and CD1a dendritic cell decreases relative to each other and controls.

An intriguing result from this study is that even tumor-free ALNs exhibited changes in immune profile, with suppression of CD4 and CD8 T cells relative to controls. In contrast, tumor-free ALNs exhibited higher dendritic cell populations than controls, and this elevation was more prominent in tumor-free ALNs from patients with positive ALNDs than from patients with negative ALNDs. This demonstrates that perturbations of the immune profile in tumor-free ALNs are dynamic and may occur before gross nodal metastasis. Our findings extend prior studies in melanoma, lung, head and neck, gastric, and breast cancer, which linked immune down-regulation only to tumor invasion, and also show that the relationship between increasing tumor invasion and changes in immune profile is not a simple linear one, as previously suggested [18,30–33].

While prognostic factors, including lymph node metastasis, tumor size, and histological grade, for breast cancer recurrence and overall survival are well established, few studies have thoroughly examined the influence of immune profile on clinical outcome [10,14,15]. To our knowledge, our findings represent the first demonstration of the clinical significance of T helper and dendritic profiles within tumor-draining nodes of breast cancer patients in predicting DFS. A recent study identified a direct relationship with SLN dendritic cell density and DFS in melanoma [34]. However, we found that the immune profile of SLNs does not display the predictive strength of ALN profiling, but rather reflects largely the metastatic status of the SLN (either tumor-involved or tumor-free). In contrast, the ALN immune profile appears much less influenced by the presence of intranodal metastatic tumor cells. We speculate that as the direct (tumor infiltration) and indirect (altered cytokine profile) effects of cancer progression alter the nodal environment, the predictive capacity of the SLN immune profile becomes diminished, and the influence of infiltrating tumor is augmented. This is analogous to observations in melanoma, in which proximity to primary tumor is the dominant determinant of immune profile [30,35,36]. By profiling ALNs, we observed a predictive accuracy of recurrence by dendritic and T cell populations that is superior even to the predictive accuracy of tumor involvement within the identical node. Furthermore, ALN immune profile predicted recurrence independent of presence or absence of metastasis on ALND. Therefore, a single axillary (nonsentinel) node, selected regardless of tumor involvement within the node or the overall status of all other nodes from the patient's ALND, contains a unique immune profile of potential prognostic value.

In summary, our findings suggest that changes in the immune profile of breast cancer-draining lymph nodes appear to accompany, and may precede, tumor invasion. Perturbation of the SLN immune profile, while highly correlated with the presence of infiltrating metastases, does not add further predictive value in patient prognosis. In contrast, our data show that ALN immune profile does predict DFS much better than it does ALN nodal metastasis. These findings raise the intriguing possibility that two independent processes may be responsible for the immune changes in sentinel versus axillary lymph nodes. The prognostic value of ALNs is highlighted by the capacity of immune profiling of a single, randomly selected ALN to stratify risk of recurrence among early stage breast cancer. Immune profiling of ALN CD4 T cells and CD1a dendritic cells among T1 and T2 tumors dramatically differentiates a population at high risk of recurrence significantly better than all available clinicopathologic patient characteristics. The additional prognostic significance of the immune profile among this subset of breast tumors is not possible by other patient, tumor, or lymph node characteristics. These observations warrant a larger, prospective confirmatory study. Our findings support that a subset of patients may be at higher risk of recurrence due to the extent of immune profile changes, and may therefore justify consideration of more aggressive therapy. Finally, our findings offer possible mechanisms underlying breast cancer's poor immunogenicity, due to either deficient co-stimulation secondary to low helper T cell populations, or inability to activate T cells as a result of down-regulation of antigen-presenting dendritic cells. Strategies to augment T cell and dendritic cell populations and function within tumor-draining nodes may increase the potential for an effective immune response and thus improve clinical outcome among breast cancer patients.

## Supporting Information

Figure S1 ALN Status, Learning Set Series 1 ALND was positive in 16 of 29 individuals. Tumor involvement was determined for a single ALN per individual (learning set ALN series 1) (*n* = 29), and nine ALNs contained tumor infiltration. Of 20 tumor-free ALNs, seven were selected from patients with a positive ALND and 13 from patients with a negative ALND.
(64 KB TIF).

Figure S2 ALN Status, Learning Set Series 2 ALND was positive in 16 of 29 individuals. Tumor involvement was determined for a single ALN per individual (learning set ALN series 2) (*n* = 27), and seven ALNs contained tumor infiltration. Of 20 tumor-free ALNs, eight were selected from patients with a positive ALND and 12 from patients with a negative ALND.
(64 KB TIF).

Figure S3 ALN Status, Test Set ALND was positive in 31 of 48 individuals. Tumor involvement was determined for a single ALN per individual (test set) (*n* = 48), and 17 ALNs contained tumor infiltration. Of 31 tumor-free ALNs, 14 were selected from patients with a positive ALND and 17 from patients with a negative ALND.
(64 KB TIF).

Figure S4 Predictive Strength of Patient and Immune Profile Characteristics, Learning Set (A) ROC curve calculating the sensitivity and specificity of lymph node CD4 T cell, CD1a dendritic cell, and ratio of CD4:CD8 T cell populations in detecting nodal metastases from the learning set (SLN, *n* = 29; ALN series 1, *n* = 29).
(B) ROC curve calculating the sensitivity and specificity of, first, primary tumor size and percent of lymph node occupied by infiltrating tumor, and second, ALN series 1 CD4 T cell, CD1a dendritic cell, and ratio of CD4:CD8 T cell populations in predicting DFS. ALN series 1 represent the first, arbitrarily selected ALN per individual in the learning set. Greater area under the curve indicates greater predictive strength. Adjusted *p*-values were determined by ROC curve testing for comparison of variable's predictive capacity.
(59 KB TIF).

**Patient Summary**

**Background**

In its earliest stage, breast cancer is confined to the breast itself, but subsequently many cancers spread to other tissues. This often happens through the lymphatic system, a set of canals similar to blood vessels that transport lymph fluid. Lymph nodes are filters along the lymphatic system. The lymph fluid draining away from the breast area is mostly filtered in a set of lymph nodes in the armpit, the so-called axillary lymph nodes. To find out whether a breast cancer has started to spread, doctors routinely check the lymph nodes for breast cancer cells that have escaped from the tumor in the breast. This used to involve surgery to remove many or all of the approximately 30 axillary lymph nodes. Because the surgery can lead to side effects like chronic pain and swelling, doctors have started more recently to first remove the “sentinel” lymph node—the first filter through which the lymph from the tumor tissue drains. In most cases, additional nodes are removed only if this first one is found to contain cancer cells.

**Why Was This Study Done?**

We know that our immune system can recognize and fight cancer cells. Cancer develops only once the immune system has been compromised, and the actual state of the immune system might tell us something about how easy and quickly the cancer will grow and spread. Because the lymph fluid contains many immune system cells, the researchers thought that (besides looking for cancer cells) it might be worth checking the lymph nodes that are closest to the tumor for immune system activity.

**What Did the Researchers Do and Find?**

They counted the numbers of different immune system cells in lymph nodes from 77 breast cancer patients. All of the patients had tumor cells in their sentinel lymph nodes, and in 42 patients tumor cells were also found in other axillary lymph nodes. For all patients, the researchers knew whether their cancers came back within five years of removing the lymph nodes. They found that the pattern of immune cells in the sentinel lymph nodes correlated with the presence of cancer cells. In the axillary lymph nodes, however, the decrease in two types of immune cells was correlated with disease-free survival regardless of the presence or absence of tumor cells in these nodes.

**What Does This Mean?**

This suggests that immune cell characteristics in axillary nodes might provide information about how likely it is that a patient's cancer comes back. These are intriguing but early results that need to be confirmed by new and larger studies before it becomes clear whether regular examination of immune system cells in lymph nodes of breast cancer patients can tell us which cancers are likely to spread and thus should be treated more aggressively.

**Where Can I Find More Information Online?**

The following Web sites contain information on the role of lymph node dissection and examination in breast cancer.

Breastcancer.org (search for “lymph node removal” and “sentinel lymph node dissection”):

http://www.breastcancer.org

People Living with Cancer (search for “sentinel lymph node biopsy” or “axillary lymph node”):

http://www.plwc.org/

Medicineworld.org (search for “axillary lymph node dissection”):

http://medicineworld.org/

## Abbreviations

ALN: axillary (nonsentinel) lymph node
ALND: axillary lymph node dissection
DFS: disease-free survival
HES: hematoxylin and eosin stained sections
IHC: immunohistochemistry
KM: Kaplan-Meier
ROC: receiver-operating-characteristic
SLN: sentinel lymph node

## References

[pmed-0020284-b01] Badwe RA, Thorat MA, Parmar VV (2003). Sentinel-node biopsy in breast cancer. N Engl J Med.

[pmed-0020284-b02] Page DL, Jensen RA, Simpson JF (1998). Routinely available indicators of prognosis in breast cancer. Breast Cancer Res Treat.

[pmed-0020284-b03] Veronesi U, Paganelli G, Viale G, Luini A, Zurrida S (2003). A randomized comparison of sentinel-node biopsy with routine axillary dissection in breast cancer. N Engl J Med.

[pmed-0020284-b04] Krag D, Weaver D, Ashikaga T, Moffat F, Klimberg VS (1998). The sentinel node in breast cancer—A multicenter validation study. N Engl J Med.

[pmed-0020284-b05] Veronesi U, Paganelli G, Galimberti V, Viale G, Zurrida S (1997). Sentinel-node biopsy to avoid axillary dissection in breast cancer with clinically negative lymph-nodes. Lancet.

[pmed-0020284-b06] Moore KH, Thaler HT, Tan LK, Borgen PI, Cody HS, 3rd (2004). Immunohistochemically detected tumor cells in the sentinel lymph nodes of patients with breast carcinoma: Biologic metastasis or procedural artifact?. Cancer.

[pmed-0020284-b07] Dowlatshahi K, Fan M, Snider HC, Habib FA (1997). Lymph node micrometastases from breast carcinoma: Reviewing the dilemma. Cancer.

[pmed-0020284-b08] Pargaonkar AS, Beissner RS, Snyder S, Speights VO (2003). Evaluation of immunohistochemistry and multiple-level sectioning in sentinel lymph nodes from patients with breast cancer. Arch Pathol Lab Med.

[pmed-0020284-b09] Weigelt B, Verduijn P, Bosma AJ, Rutgers EJ, Peterse HL (2004). Detection of metastases in sentinel lymph nodes of breast cancer patients by multiple mRNA markers. Br J Cancer.

[pmed-0020284-b10] Coventry BJ, Morton J (2003). CD1a-positive infiltrating-dendritic cell density and 5-year survival from human breast cancer. Br J Cancer.

[pmed-0020284-b11] Coventry BJ (1999). CD1a positive putative tumour infiltrating dendritic cells in human breast cancer. Anticancer Res.

[pmed-0020284-b12] Georgiannos SN, Renaut A, Goode AW, Sheaff M (2003). The immunophenotype and activation status of the lymphocytic infiltrate in human breast cancers, the role of the major histocompatibility complex in cell-mediated immune mechanisms, and their association with prognostic indicators. Surgery.

[pmed-0020284-b13] Liyanage UK, Moore TT, Joo HG, Tanaka Y, Herrmann V (2002). Prevalence of regulatory T cells is increased in peripheral blood and tumor microenvironment of patients with pancreas or breast adenocarcinoma. J Immunol.

[pmed-0020284-b14] Iwamoto M, Shinohara H, Miyamoto A, Okuzawa M, Mabuchi H (2003). Prognostic value of tumor-infiltrating dendritic cells expressing CD83 in human breast carcinomas. Int J Cancer.

[pmed-0020284-b15] Lespagnard L, Gancberg D, Rouas G, Leclercq G, de Saint-Aubain Somerhausen N (1999). Tumor-infiltrating dendritic cells in adenocarcinomas of the breast: A study of 143 neoplasms with a correlation to usual prognostic factors and to clinical outcome. Int J Cancer.

[pmed-0020284-b16] Blake-Mortimer JS, Sephton SE, Carlson RW, Stites D, Spiegel D (2004). Cytotoxic T lymphocyte count and survival time in women with metastatic breast cancer. Breast J.

[pmed-0020284-b17] McDermott RS, Beuvon F, Pauly M, Pallud C, Vincent-Salomon A (2002). Tumor antigens and antigen-presenting capacity in breast cancer. Pathobiology.

[pmed-0020284-b18] Laguens G, Coronato S, Laguens R, Portiansky E, Di Girolamo V (2002). Human regional lymph nodes draining cancer exhibit a profound dendritic cell depletion as comparing to those from patients without malignancies. Immunol Lett.

[pmed-0020284-b19] Alam SM, Clark JS, George WD, Campbell AM (1993). Altered lymphocyte populations in tumour invaded nodes of breast cancer patients. Immunol Lett.

[pmed-0020284-b20] Poindexter NJ, Sahin A, Hunt KK, Grimm EA (2004). Analysis of dendritic cells in tumor-free and tumor-containing sentinel lymph nodes from patients with breast cancer. Breast Cancer Res.

[pmed-0020284-b21] Dunn O, Clark V (2000). Basic statistics: A primer for biomedical sciences, 3rd Ed.

[pmed-0020284-b22] Heidenreich W, Jagla K, Schussler J, Borner P, Dehnhard F (1979). Immunological characterization of mononuclear cells in peripheral blood and regional lymph nodes of breast cancer patients. Cancer.

[pmed-0020284-b23] Bryan CF, Eastman PJ, Conner JB, Baier KA, Durham JB (1993). Clinical utility of a lymph node normal range obtained by flow cytometry. Ann N Y Acad Sci.

[pmed-0020284-b24] Vidal-Rubio B, Sanchez-Carril M, Oliver-Morales J, Gonzalez-Femandez A, Gambon-Deza F (2001). Changes in human lymphocyte subpopulations in tonsils and regional lymph nodes of human head and neck squamous carcinoma compared to control lymph nodes. BMC Immunol.

[pmed-0020284-b25] Singletary SE, Greene FL, Sobin LH (2003). Classification of isolated tumor cells: Clarification of the 6th edition of the American Joint Committee on Cancer Staging Manual. Cancer.

[pmed-0020284-b26] Zhang L, Conejo-Garcia JR, Katsaros D, Gimotty PA, Massobrio M (2003). Intratumoral T cells, recurrence, and survival in epithelial ovarian cancer. N Engl J Med.

[pmed-0020284-b27] Ihaka R, Gentleman R (1996). R: A Language for Data Analysis and Graphics. J Comp Graph Stat.

[pmed-0020284-b28] Maindonald J, Braun J (2003). Data analysis and graphics using R.

[pmed-0020284-b29] Dowlatshahi K, Fan M, Bloom KJ, Spitz DJ, Patel S (1999). Occult metastases in the sentinel lymph nodes of patients with early stage breast carcinoma: A preliminary study. Cancer.

[pmed-0020284-b30] Cochran AJ, Pihl E, Wen DR, Hoon DS, Korn EL (1987). Zoned immune suppression of lymph nodes draining malignant melanoma: Histologic and immunohistologic studies. J Natl Cancer Inst.

[pmed-0020284-b31] Battaglia A, Ferrandina G, Buzzonetti A, Malinconico P, Legge F (2003). Lymphocyte populations in human lymph nodes. Alterations in CD4+ CD25+ T regulatory cell phenotype and T-cell receptor Vbeta repertoire. Immunology.

[pmed-0020284-b32] Shinkal H, Kitayama J, Kimura W, Muto T, Shibata Y (1996). Functional expression of CD11a on CD8+ cells is suppressed in regional lymph nodes with cancer involvement in patients with gastrointestinal carcinoma. Cancer.

[pmed-0020284-b33] Takenoyama M, Yasumoto K, Harada M, Matsuzaki G, Ishida T (1996). Expression of activation-related molecules on regional lymph node lymphocytes in human lung cancer. Immunobiology.

[pmed-0020284-b34] Cochran AJ, Wen DR, Huang RR, Wang HJ, Elashoff R (2004). Prediction of metastatic melanoma in nonsentinel nodes and clinical outcome based on the primary melanoma and the sentinel node. Mod Pathol.

[pmed-0020284-b35] Cochran AJ, Morton DL, Stern S, Lana AM, Essner R (2001). Sentinel lymph nodes show profound downregulation of antigen-presenting cells of the paracortex: Implications for tumor biology and treatment. Mod Pathol.

[pmed-0020284-b36] Essner R, Kojima M (2002). Dendritic cell function in sentinel nodes. Oncology (Huntingt).

